# NLRP3 Inflammasome Activation in the Brain after Global Cerebral Ischemia and Regulation by 17*β*-Estradiol

**DOI:** 10.1155/2016/8309031

**Published:** 2016-10-23

**Authors:** Roshni Thakkar, Ruimin Wang, Gangadhara Sareddy, Jing Wang, Dharma Thiruvaiyaru, Ratna Vadlamudi, Quanguang Zhang, Darrell Brann

**Affiliations:** ^1^Department of Neuroscience and Regenerative Medicine, Medical College of Georgia, Augusta University, Augusta, GA, USA; ^2^Department of Obstetrics and Gynecology, University of Texas Health Science Center, San Antonio, TX, USA; ^3^Department of Mathematics, Augusta University, Augusta, GA, USA

## Abstract

17*β*-Estradiol (E2) is a well-known neuroprotective factor in the brain. Recently, our lab demonstrated that the neuroprotective and cognitive effects of E2 require mediation by the estrogen receptor (ER) coregulator protein and proline-, glutamic acid-, and leucine-rich protein 1 (PELP1). In the current study, we examined whether E2, acting via PELP1, can exert anti-inflammatory effects in the ovariectomized rat and mouse hippocampus to regulate NLRP3 inflammasome activation after global cerebral ischemia (GCI). Activation of the NLRP3 inflammasome pathway and expression of its downstream products, cleaved caspase-1 and IL-1*β*, were robustly increased in the hippocampus after GCI, with peak levels observed at 6-7 days. Expression of P2X7 receptor, an upstream regulator of NLRP3, was also increased after GCI. E2 markedly inhibited NLRP3 inflammasome pathway activation, caspase-1, and proinflammatory cytokine production, as well as P2X7 receptor expression after GCI (at both the mRNA and protein level). Intriguingly, the ability of E2 to exert these anti-inflammatory effects was lost in PELP1 forebrain-specific knockout mice, indicating a key role for PELP1 in E2 anti-inflammatory signaling. Collectively, our study demonstrates that NLRP3 inflammasome activation and proinflammatory cytokine production are markedly increased in the hippocampus after GCI, and that E2 signaling via PELP1 can profoundly inhibit these proinflammatory effects.

## 1. Introduction

The steroid hormone, 17*β*-estradiol (E2), is produced primarily by the ovaries in females and has multiple actions throughout the body and brain. In the brain, there is a significant body of work demonstrating that E2 is neuroprotective in both acute and chronic neurodegenerative disorders such as cerebral ischemia, traumatic brain injury, Alzheimer's disease, and Parkinson's disease [1–4]. For instance, in 1997, Simpkins et al. demonstrated for the first time that E2 was neuroprotective in an ovariectomized rat focal cerebral ischemia (FCI) model [5]. Since then, numerous studies have confirmed the neuroprotective effects of E2 in both focal and global cerebral ischemia (GCI) models and implicated both genomic and extranuclear signaling mechanisms in mediation of the E2 effects [4, 6–11]. In the brain, the neuroprotective actions of E2 have been implicated to be mediated by the classical estrogen receptors, estrogen receptor-*α* (ER-*α*) and estrogen receptor-*β* (ER-*β*) [4, 9]. However, there is also evidence that a new putative ER called G-protein coupled receptor 1 (GPER1) may also contribute to neuroprotection [6, 12, 13].

Upon E2 binding to ER, several coregulatory proteins associate with the ER to regulate E2 transcriptional activity. The ER-coregulator protein association leads to formation of an “ER signalosome”, which facilitates both ER genomic and extranuclear actions [14]. Recent work by our group led to the cloning and characterization of a novel ER coregulator, called proline-, glutamic acid-, and leucine-rich protein 1 (PELP1) [15, 16]. PELP1 is a multidomain scaffold protein that can interact with ERs using the nuclear receptor interaction motif, LXXLL, and with Src kinase and PI3K kinase using SH2 and PXXP motifs [16]. PELP1 is expressed in a variety of tissues, with highest expression in the brain, ovaries, testes, and uterus [16–20]. In addition, PELP1 exhibits both nuclear and cytoplasmic localization [20], and phosphorylation of PELP1 can control its localization, interaction with other proteins, and stability in cells [16, 17]. To understand its importance in E2 actions in the brain, our group recently created a PELP1 forebrain-specific knockout (PELP1 FBKO) mouse model [16]. Using the PELP1 FBKO mouse model, we demonstrated a critical role for PELP1 in mediating E2 regulation of extranuclear and genomic signaling, as well as E2-induced neuroprotection and cognitive function in the hippocampus following ischemic injury [16].

While the role of E2 as a neuroprotective factor has been well established, its role in regulation of neuroinflammation has been less studied. Growing evidence suggests that neuroinflammation can contribute significantly to neuronal damage and cell death in neurodegenerative disorders, and that attenuation of neuroinflammation can be neuroprotective [21–23]. With respect to E2, several studies have shown that it can suppress microglial activation and proinflammatory cytokine production* in vivo* and* in vitro* [24–28], although the mechanisms underlying the effects remain unclear. A significant advance to the neuroinflammation field occurred in 2002, when Martinon and coworkers identified “inflammasomes” as key proteins that trigger the inflammatory response [29]. Inflammasomes are multiprotein complexes that mediate activation of caspase-1 and promote secretion of proinflammatory cytokines. Inflammasomes are activated in response to either pathogen associated molecular patterns (PAMPs), derived from invading pathogens, or damage-associated molecular patterns (DAMPs), released by dying cells [30]. Inflammasomes belong to the NOD-like receptor family (NLRs). NLRs are encoded by a family of 22 genes and are mainly divided as NLRP (NOD-like receptor with a pyrin domain) and NLRC (NOD-like receptors with CARD domain) family members [31].

The structure of NLRP includes a carboxy-terminal leucine rich repeat (LRR), a nucleotide binding domain called the NACHT domain, and an N-terminal pyrin domain (PYD). The PYD domain of inflammasome binds to a PYD domain of apoptosis associated speck like protein (ASC), which is an adaptor protein [31]. ASC also has a caspase activation and recruitment (CARD) domain, which binds to the CARD domain of the procaspase-1 enzyme. NLRP3 is the most abundantly found inflammasome and contributes to the majority of production of proinflammatory cytokines [23, 31, 32]. Inflammasomes are primed and activated by triggers like pathogens and metabolic and genotoxic stressors. For instance, the P2X7 receptor is activated by elevation of extracellular ATP, which then leads to activation of the NLRP3 inflammasome complex [33]. Once activated, inflammasomes enhance downstream activation of caspase-1 and release of proinflammatory cytokines, such as IL-1*β* and IL-18 [29, 34], which initiate a cascade of detrimental inflammatory events and apoptosis [35].

Of all of the inflammasome proteins, NLRP3 is by far the most studied inflammasome in cerebral ischemia studies. NLPR3 expression has been described in multiple brain cell types, including astrocytes, microglia, neurons, and endothelial cells [22, 36–39]. Numerous studies have shown that NLRP3 is elevated in the brain of human stroke patients [40] and experimental stroke animals [40–44]. Furthermore, NLRP3 appears to have an important role in ischemic pathology as NLRP3 knockout animals have significantly reduced infarct size and neurovascular damage after focal cerebral ischemia [38]. Additionally, immunoglobulin treatment suppresses NLRP3 activity and strongly protects neurons in ischemic animals [40]. In addition, Aim2 and NLRC4 knockout animals have also been reported to have reduced inflammation and infarct size after focal cerebral ischemia, but the effects appear to be independent of IL-1*β* production [45].

With respect to E2 regulation of NLRP3 inflammasome activation, E2 has been reported in one study to suppress NLRP3 inflammasome gene expression in the cerebral cortex after focal cerebral ischemia (FCI) [46], while in a second study an ER-*β* agonist suppressed caspase-1, ASC, and IL-1*β* expression in the hippocampus after global cerebral ischemia (GCI) [47]. These studies suggest that E2 can regulate inflammasome activation; however, several important questions remain unanswered, including the following: (1) What is the temporal pattern of NLRP3 inflammasome expression in the hippocampus after GCI? (2) In which cell type in the hippocampus does NLRP3 inflammasome activation occur? (3) Does E2 only affect expression of the NLRP3 inflammasome or does it also regulate NLRP3 inflammasome complex formation? (4) What are the mechanisms underlying E2 regulation of the NLRP3 inflammasome? (5) Does the ER coregulator, PELP1, mediate E2 regulation of the NLRP3 inflammasome in the brain? The goal of the current study was to address these key questions. The results of the study reveal that both expression and complex formation of the NLRP3 inflammasome, as well as activated caspase-1 and IL-1*β*, are robustly increased in the hippocampus of both the rat and mouse after GCI, and this effect is strongly inhibited by E2 replacement. The results also demonstrate that NLRP3 inflammasome activation occurs in both microglia and astrocytes after GCI, and that E2 inhibits expression of both P2X7 and TXNIP, two well-known upstream inducers of NLRP3 inflammasome activation. Finally, the results reveal that the ER coregulator protein PELP1 is essential for mediation of E2 regulatory effects upon activation of the NLRP3 inflammasome.

## 2. Materials and Methods

### 2.1. Animals and Surgical Procedures

#### 2.1.1. Ovariectomized Rat Studies

Augusta University Institutional Animal Care and Use Committee approved all animal procedures and the studies were conducted in accordance with National Institutes of Health guidelines for animal research. Three-month-old young adult Sprague Dawley rats were bilaterally ovariectomized under isoflurane anesthesia and separated into shams, ischemia-reperfusion injury, and injury with estrogen (E2) treatment groups. The E2 treatment group animals were immediately administered with 17*β*-estradiol dissolved in 20%  *β*-cyclodextrin added to mini-pumps (0.5 *μ*L/hr, 14-day release; Alzet, Cupertino, CA). Pumps were placed in the upper mid-back region to allow subcutaneous administration of E2. The dose of E2 used led to production of physiological Diestrus I levels of circulating E2 (10–15 pg/mL). All rats, except for sham controls, were subjected to global cerebral ischemia (GCI) via 4-vessel occlusion method [48] after 7 days of ovariectomy. One day prior to occlusion, that is, 6 days after ovariectomy, all animals were anesthetized using ketamine/xylazine and their vertebral arteries were electrocauterized and the common carotid arteries were exposed. Twenty-four hours later, the common carotid arteries were transiently occluded with hemostatic clips for 12 minutes for all animals except the shams. Sham animals had their arteries exposed but not occluded. Ischemia-reperfusion was allowed to occur and animals were sacrificed using transcardial perfusion and decapitation at 1, 3, and 7 days after GCI.

#### 2.1.2. PELP1 Forebrain-Specific Knockout Mouse Studies

Young adult female C27BL/6 PELP1 forebrain-specific knockout mice were generated as described by our group previously [16]. FLOX control as well as PELP1 knockout mice were bilaterally ovariectomized and implanted with either placebo or 17*β*-estradiol (E2) subcutaneous mini-pumps immediately following ovariectomy. After 7 days of ovariectomy, all animals except the shams were subjected to two-vessel occlusion for global cerebral ischemia (GCI). Animals were anesthetized briefly with ketamine/xylazine and the two common carotid arteries were exposed and occluded transiently with hemostatic clips for 40 minutes. Sham animals had their arteries exposed but not occluded. All animals were sacrificed using transcardial perfusion at 6 days after GCI.

### 2.2. Tissue Collection

All animals were transcardially perfused and decapitated at the desired time point after GCI. Brains were dissected in the midsagittal plane and fixed in 4% paraformaldehyde for 24 hours, cryoprotected in 30% sucrose, and sectioned on a cryostat to obtain 20-micron-thick hippocampal sections. These sections were then used for immunofluorescence staining and proximity ligation assay (Duolink). For RT-PCR and Western blot analysis, brains were collected and the hippocampal tissue was dissected out, frozen, and either processed for RNA isolation via one-step RT-PCR or homogenized for detection of proteins via Western blot analysis as described below.

### 2.3. RT-PCR

Hippocampal tissue samples were collected and RNA was isolated using the SV total RNA isolation system (Promega). The RNA was then used for the reverse transcriptase PCR reaction using the Superscript III one-step RT-PCR system with platinum Taq DNA Polymerase (Invitrogen) and the primers listed in Table 1 (Integrated DNA Technologies). The gene expression analyses were done using the comparative ΔΔCt method. The mRNA level changes were expressed as fold change as compared to the sham animals. All Ct values for target genes were normalized to CypA gene [49].

### 2.4. Western Blot Analysis

Hippocampal tissue was collected 7 days after GCI as mentioned above. Individual hippocampal sample from each animal was homogenized in RIPA buffer using silica beads and a Mini-Beadbeater (Biospec Products, OK, USA). The homogenate was centrifuged at 13,000 rpm for 10 minutes at 4°C  and the supernatant was used for protein estimation by Lowry's Assay (Lowry's Assay Kit, Sigma). Fifty micrograms of protein for each sample was separated on 15% sodium dodecyl sulfate-polyacrylamide gel electrophoresis, transferred on nitrocellulose membrane, and blocked with 5% bovine serum albumin for 1 hour at room temperature with gentle shaking. Blocking was followed by incubation with primary antibodies, NLRP3 (Santa-Cruz Biotechnology, sc-34408), ASC (Santa-Cruz Biotechnology, sc-22514-R), cl-caspase-1 (Santa-Cruz Biotechnology, sc-22165), and IL-1*β* (Abcam, ab9722), overnight at 4°C with gentle shaking. Glyceraldehyde 3-phosphate dehydrogenase (GAPDH, Santa-Cruz Biotechnology, sc-32233) was used as a loading control. The membrane was then washed with 1x TBST to remove unbound primary antibody and incubated with secondary Alexa Fluor 680 or 800 anti-rabbit/goat/mouse IgG for 1 hour at room temperature with gentle shaking. Blots were scanned using Odyssey Imaging System (LI-COR Bioscience, Lincoln, NB). The intensity of bands was quantified using ImageJ software. The immunoblot data was corrected for corresponding GAPDH values and presented as fold change in protein as compared to sham animals.

### 2.5. Immunofluorescence Staining and Confocal Microscopy Analysis

Twenty *μ*m thick coronal sections were washed with PBS and 0.4% Triton-X PBS for 20 minutes. The sections were then blocked with 10% normal donkey serum for 1 hour at room temperature in PBS containing 0.1% Triton X-100, followed by incubation with primary antibody for 1–3 nights at 4°C in the same buffer. Primary antibodies used for this study included rabbit-NLRP3 (Santa-Cruz Biotechnology, sc-66846), rabbit-ASC (Santa-Cruz Biotechnology, sc-22514-R), goat-cleaved caspase-1 (Santa-Cruz Biotechnology, sc-22165), rabbit-IL-1*β* (Abcam, ab9722), goat-Iba1 (Abcam, ab5076), rabbit-P2X7 receptor (Sigma, P8232), and mouse-NeuN (Millipore, MAB377). After primary antibody incubation, sections were washed for 3 × 10 minutes at room temperature (RT), followed by incubation with the appropriate secondary antibody: Alexa-Fluor488/568/647 donkey anti-rabbit/anti-mouse/anti-goat (Invitrogen) RT/1 hour. Sections were then washed with PBS containing 0.1% Triton X-100 for 3 × 10 min, followed by 2 × 5 min with 1x PBS and briefly with water. The sections were then mounted with water-based mounting medium containing antifading agents and observed using confocal microscopy. All images were captured on a confocal laser microscope (Carl Zeiss, Germany) using the Zen software at 40x magnification and 50 *μ*m scale bar.

### 2.6. Duolink Proximity Ligation Assay

Tissue sections were blocked in 5% (vol/vol) donkey serum for 1 hour at room temperature and incubated overnight with primary antibodies, goat-NLRP3 (Santa-Cruz Biotechnology, sc-34408), and rabbit-ASC (Santa-Cruz Biotechnology, sc-22514-R) at 4°C. These sections were then incubated with Duolink PLA probes, anti-Rabbit MINUS (Sigma-Aldrich, DUO92005) and anti-goat PLUS (Sigma-Aldrich, DUO92003) for 1 h at 37°C. Ligation and amplification were carried out at 37°C using the Duolink* in situ* detection reagent kit (Sigma-Aldrich, DUO92008) according to the manufacturer's protocol. All sections were then mounted on a slide and all images were captured on a confocal laser microscope (Carl Zeiss, Germany) using the Zen software at 40x magnification and 50 *μ*m scale bar.

### 2.7. Antibody Specificity in Immunofluorescence Staining

Specificity of inflammasome pathway factor antibodies used in immunofluorescence staining was tested by preabsorption with the antigen peptide or recombinant protein (IL-1*β*) available from the manufacturer. For preabsorption, fivefold concentration of each peptide, NLRP3, ASC, cleaved caspase-1, and IL-1*β* was incubated with primary antibody (at concentrations used for immunofluorescence staining) overnight at 4°C. The immunofluorescence staining (with DAPI) was then performed as described above, and images were captured on a confocal laser microscope (Carl Zeiss, Germany) using the Zen software at 40x magnification and 50 *μ*m scale bar.

### 2.8. Quantification of Confocal Images

The intensity of all confocal images captured at 40x magnification was quantified using MATLAB software (version R2013a by Mathworks, Natick, MA, USA) as described previously [50]. MATLAB is a programming environment with built-in image processing tools. The intensity threshold for injured animals was identified by applying a multilevel image threshold algorithm using Otsu's method in MATLAB [50]. This value was then used as an intensity threshold for sham and E2-treated animals. The algorithm digitized each image into a 1024 × 1024 matrix. The individual values contained in the matrix represented the intensity value of pixels of a particular color, that is, red, blue, or green. Using the threshold value obtained from the algorithm, the image was segmented into two regions: one above the threshold value and one below. Finally, dividing the segmented area with intensity above the threshold value by the total image area enabled image quantification. The data were obtained as relative area of fluorescence as compared to the entire area of the image. The data was expressed as percentage of area activated in the entire captured field. The quantification of immunofluorescence images was also confirmed by using ImageJ intensity analysis.

### 2.9. Statistical Analysis

The independent two-sample *t*-test was conducted to investigate whether the mean fold change for mRNA of factors under study for each of the three time points (1, 3, and 7 days) was significantly different between the ischemic and the estrogen-treated rats. The one-way ANOVA test was conducted to investigate whether there is a significant difference among sham, ischemic, and E2-treated rats on day 7 after injury for all proteins under study. The two-way ANOVA test was conducted to investigate whether there is a significant difference among sham, ischemic, and E2-treated groups of FLOX control versus the PELP1 KO mice, as well as whether there is a significant interaction between group (sham, ischemic, and E2) and type (FLOX and KO) for all molecules under study. Whenever the difference for either of the ANOVA tests was found to be significant,* post hoc* tests such as Bonferroni's test or Tamhane's test was conducted to make pairwise comparisons of the groups of animals. All tests were conducted at a 5% level of significance using the IBM SPSS software (version 23).

## 3. Results

### 3.1. Temporal Expression of NLRP3 Inflammasome Pathway Factors in the Hippocampus after GCI and Regulation by Estradiol

**Figure 1:**
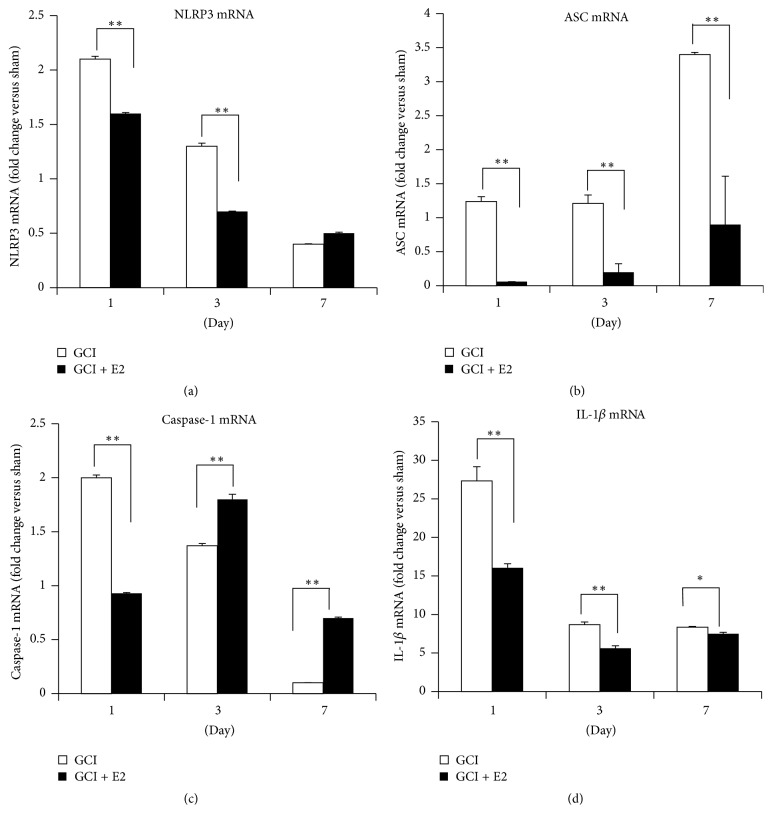
Temporal pattern of NLRP3, ASC, caspase-1, and IL-1*β* gene expression after GCI and their suppression by E2 treatment. mRNA samples were collected from the hippocampus of adult female ovariectomized rats, with and without E2, at various times after GCI. (a) NLRP3, (b) ASC, (c) caspase-1, and (d) IL-1*β* are all expressed temporally after GCI and their expression is robustly suppressed by E2 treatment at early time points after GCI (^*∗∗*^ *p* ≤ 0.0001, ^*∗*^ *p* < 0.05, GCI versus GCI + E2) (*n* = 5-6 animals per group).

**Figure 2:**
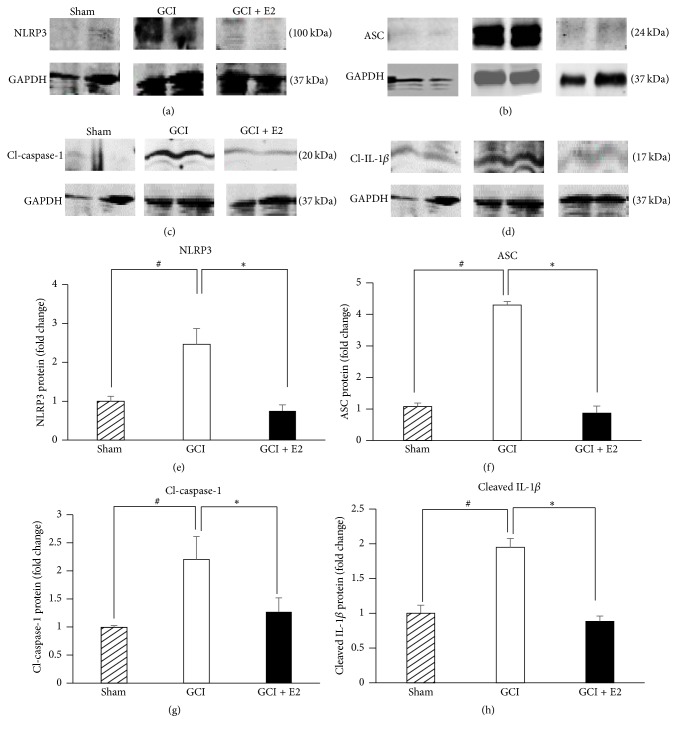
Increase in NLRP3, ASC, cleaved caspase-1, and cleaved IL-1*β* protein levels after GCI and its suppression by E2. Representative immunoblots from Western blot analysis of hippocampal samples collected 7 days after GCI show that (a) NLRP3, (b) ASC, (c) cleaved caspase-1, and (d) cleaved IL-1*β* are robustly increased after GCI as compared to shams and are significantly suppressed by E2 treatment. (e–h) Quantification of immunoblots using ImageJ software showed that NLRP3, cleaved caspase-1, and cleaved IL-1*β* levels are increased 2.5-fold after GCI and ASC is increased 6-fold. This increase in protein levels is significantly suppressed by E2 treatment (^#^ *p* < 0.05 sham versus GCI; ^*∗*^ *p* < 0.05 GCI versus GCI + E2) (*n* = 4-5 animals per group).

We first examined the temporal expression of NLRP3 inflammasome pathway factors and their regulation by E2 in the hippocampus after GCI. The first approach involved determining the temporal pattern of gene expression of inflammasome pathway molecules, NLRP3, ASC, caspase-1, and IL-1*β*. As shown in Figure 1, mRNA levels for these markers were detected using RT-PCR analysis from hippocampus tissue collected at days 1, 3, and 7 after GCI. The data are represented in terms of fold change as compared to the sham animals that did not undergo ischemia. The mRNA levels for NLRP3 (Figure 1(a)) were increased 2-fold and 1.5-fold at days 1 and 3, respectively, after injury. E2 treatment strongly suppressed NLRP3 gene expression at both days 1 and 3 after injury. Likewise, ASC mRNA levels were strongly elevated at days 1, 3, and 7 after GCI, and this effect was strongly inhibited by E2 treatment (Figure 1(b)). Caspase-1 mRNA levels were increased 2-fold at day 1 after injury and were significantly suppressed by E2 (Figure 1(c)). However, at days 3 and 7 after GCI, E2 paradoxically increased caspase-1 mRNA levels. It should be noted that the caspase-1 mRNA measured in this study represents expression of the procaspase-1, and that cleavage of the procaspase-1 protein is required for its activation. The effects of GCI and E2 upon the cleaved (active form of) caspase-1 are described below in a subsequent figure. Finally, IL-1*β* mRNA levels showed very high induction in the hippocampal CA1 region after GCI, with an 8–27-fold increase on days 1, 3, and 7 after GCI (Figure 1(d)). Similar to the results for the other NLRP3 inflammasome factors, E2 treatment robustly suppressed IL-1*β* mRNA levels at all three days after GCI. We next examined GCI and E2 regulation of protein levels of the NLRP3 inflammasome pathway molecules in the hippocampus using Western blot analysis. Representative Western blot results are shown in Figures 2(a)–2(d), while quantification of the results from all samples is shown in Figures 2(e)–2(h). As shown in Figures 2(a)–2(h), the results demonstrate that protein levels of NLRP3, ASC, and “active” cleaved caspase-1 and cleaved IL-1*β* are significantly increased in the hippocampus at 7 days after GCI, and that E2 treatment significantly attenuates the elevation of the NLRP3 inflammasome proteins.

**Figure 3:**
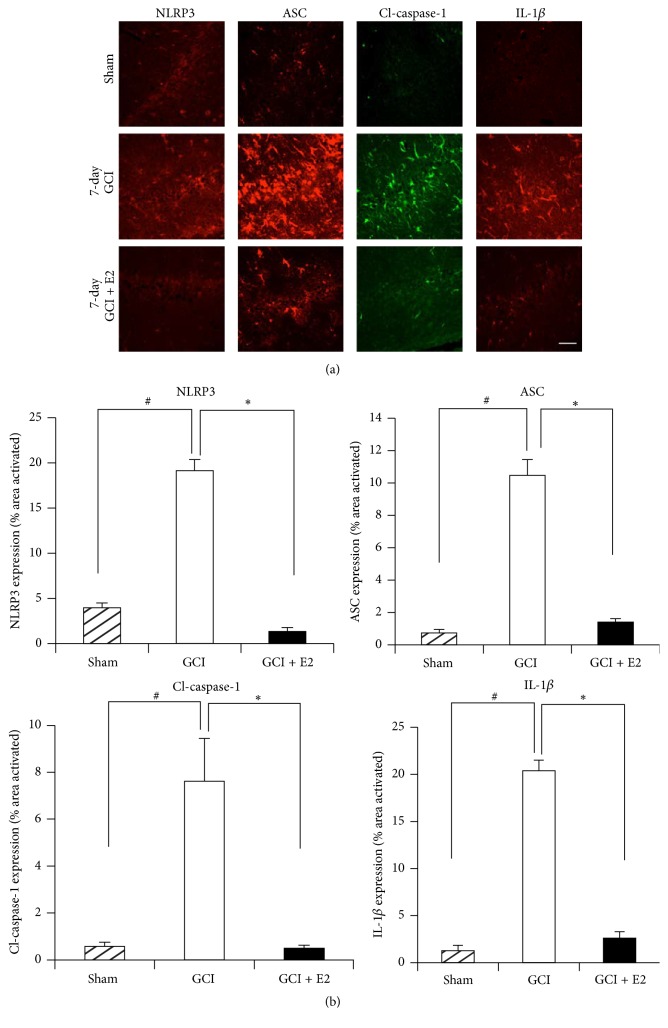
Expression of ASC, NLRP3, cleaved caspase-1, and IL-1*β* protein after GCI and suppression by E2. (a) Representative confocal images show that ASC, NLRP3, cleaved caspase-1, and IL-1*β* are activated in hippocampal CA1 region of the young adult female rat at day 7 after GCI. This expression is robustly suppressed by E2, as seen in the lower panel by reduced staining (magnification = 40x, scale bar = 50 *μ*m). (b) Quantification of fluorescence intensity using MATLAB software shows a statistically significant suppression of all of these proteins at day 7 after GCI (^#^ *p* < 0.05 sham versus GCI; ^*∗*^ *p* < 0.05 GCI versus GCI + E2) (*n* = 5-6 animals per group).

To further confirm the mRNA as well as Western blot results, we used immunofluorescence staining to detect the protein expression of NLRP3, ASC, “active” cleaved caspase-1 (cl-caspase-1), and IL-1*β* at 7 days after GCI. As shown in Figure 3(a), representative photomicrographs reveal that the expression of NLRP3, ASC, “active” cleaved caspase-1, and IL-1*β* was significantly increased in the hippocampal CA1 region of the rat hippocampus at 7 days after GCI, as compared to the sham controls. Furthermore, E2 treatment robustly suppressed the enhanced protein expression of NLRP3, ASC, “active” cleaved caspase-1, and IL-1*β* in the hippocampus after GCI.  Figure 3(b) shows the results of quantification of the immunofluorescence staining intensity using MATLAB software. As shown in Figure 3(b), all of the NLRP3 inflammasome pathway molecules were highly expressed at day 7 after GCI and were significantly suppressed by E2. The specificity of the NLRP3 inflammasome pathway antibodies used in our studies was confirmed by preabsorption studies with antigen (or when not available, with the recombinant protein, e.g., for IL-1*β*). As shown in Supplementary Figure 1 (see Supplementary Material available online at http://dx.doi.org/10.1155/2016/8309031), antigen or recombinant protein preabsorption essentially completely abolished staining for the NLRP3 inflammasome molecules, demonstrating specificity of the antibodies. Therefore, coupled with the mRNA expression and Western blot data in Figures 1 and 2, the results in Figure 3 further confirm that expression of NLRP3 inflammasome pathway molecules is increased in the rat hippocampus after GCI and suppressed by E2 treatment.

Using triple immunofluorescent staining, we next determined the cell type of expression of NLRP3 inflammasome molecules in the hippocampal CA1 region at 7 days after GCI. As shown in Supplementary Figure 2, triple immunofluorescence staining of each of the NLRP3 inflammasome pathway molecules with either GFAP (astrocyte marker) or CD11b (microglial marker) and NeuN (neuronal marker) or DAPI revealed that NLRP3, ASC, “active” cleaved caspase-1, and IL-1*β* are colocalized with CD11b and GFAP in the hippocampal CA1 region at 7 days after GCI. This finding suggests that NLRP3 inflammasome pathway activation occurs primarily in microglia and activated astrocytes in the hippocampus at 7 days after GCI.

### 3.2. Estradiol Suppresses NLRP3 Inflammasome Complex Formation in the Hippocampus after GCI

**Figure 4:**
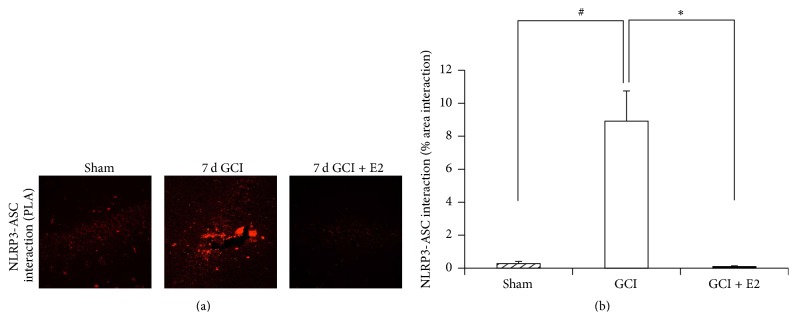
Proximity ligation assay demonstrating NLRP3-ASC complex formation after GCI and its suppression by E2. (a) Representative confocal images of proximity ligation assay (Duolink* in situ* co-IP) show that NLRP3-ASC complex is formed in hippocampal CA1 region of the young adult female rat at day 7 after GCI. This complex formation is robustly suppressed by E2 (magnification = 40x, scale bar = 50 *μ*m). (b) Quantification of fluorescence intensity using MATLAB software shows a statistically significant suppression of complex formation at day 7 after GCI (^#^ *p* < 0.05 sham versus GCI; ^*∗*^ *p* < 0.05 GCI versus GCI + E2) (*n* = 5-6 animals per group).

In the above studies, we examined E2 regulation of expression of NLRP3 inflammasome pathway factors after GCI. To further confirm activation of the NLRP3 inflammasome, we examined NLRP3 inflammasome* complex formation* after GCI, as assembly of the inflammasome complex is known to be essential for its activation. To examine NLRP3 inflammasome complex formation, we performed an* in situ* coimmunoprecipitation assay, also known as a proximity ligation assay (or Duolink assay), to measure the protein-protein interaction (complex formation) of NLRP3 and ASC in the rat hippocampal CA1 region at 7 days after GCI. ASC binding to NLRP3 is known to be critical for recruitment of caspase-1 and for NLRP3 inflammasome activation [31]. The protein-protein interaction in the proximity ligation assay was detected as red immunofluorescence signal under a confocal microscope. Representative photomicrographs of the proximity ligation assay results are shown in Figure 4(a), while quantification of the results from all images from all animals for intensity using MATLAB is shown in Figure 4(b). As shown in Figures 4(a) and 4(b), the results of the proximity ligation assay revealed a significant increase in NLRP3-ASC complex formation in the hippocampus after GCI, as compared to sham controls. Furthermore, E2 treatment strongly suppressed the GCI-induced NLRP3-ASC complex formation in the hippocampus.

### 3.3. Estradiol Regulates Expression of Upstream Activators of the NLRP3 Inflammasome

**Figure 5:**
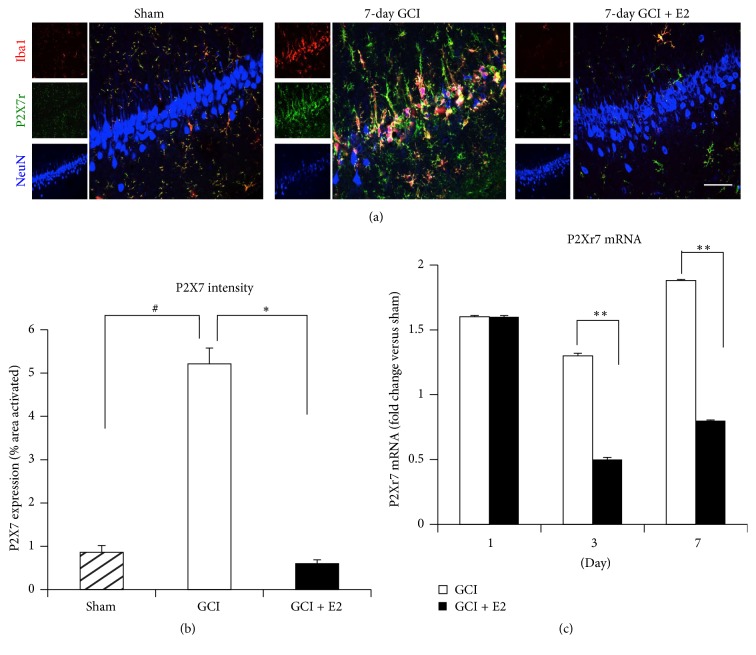
Increased expression of P2X7 receptor after GCI and its suppression by E2 treatment. (a) Representative confocal images stained for Iba1 (red), P2X7r (green), and NeuN (blue) show the activation of microglia and increased expression of P2X7r in the hippocampal CA1 region of young adult female rats 7 days after GCI. The P2X7 receptor colocalized very strongly with microglia (yellow color). This expression and microglial activation is suppressed by E2 treatment (magnification = 40x, scale bar = 50 *μ*m). (b) Quantification of fluorescence intensity using MATLAB software shows a statistically significant suppression of P2X7 receptor by E2 at day 7 after GCI (^#^ *p* < 0.05 sham versus GCI; ^*∗*^ *p* < 0.05 GCI versus GCI + E2) (*n* = 5-6 animals per group). (c) mRNA collected from rat hippocampus after GCI shows induction of P2rX7 gene and its significant suppression by E2 treatment at days 3 and 7 after GCI (^*∗∗*^ *p* < 0.0001, GCI versus GCI + E2) (*n* = 5-6 animals/group).

We hypothesized that E2 may control NLRP3 inflammasome activation by regulating the expression of key upstream activators of the NLRP3 inflammasome. A well-known upstream activator of the NLRP3 inflammasome is the ionotropic purinergic P2X7 receptor. The P2X7 receptor becomes activated by extracellular ATP, which is a danger-associated molecular pattern (DAMP) released from damaged/dying neurons after injury [51]. We thus examined the ability of E2 to regulate protein and mRNA expression levels of the P2X7 receptor in the hippocampus using triple immunofluorescent staining and quantitative RT-PCR. Representative photomicrographs of the triple immunofluorescent staining results for P2X7 receptor, NeuN (neuronal marker), and Iba1 (microglial marker) are shown in Figure 5(a), while quantification of the results from all images from all animals for intensity using MATLAB is shown in Figure 5(b). As shown in Figures 5(a) and 5(b), triple immunofluorescence colocalization results with specific antibodies to the P2X7 receptor, NeuN (neuronal marker), and Iba1 (microglial marker) revealed that immunoreactive protein levels of the P2X7 receptor in hippocampal CA1 region of rat are strongly upregulated at 7 days after GCI. Furthermore, the P2X7 receptor immunostaining signal was highly colocalized with the microglia marker Iba1, indicating its upregulation occurs in microglia. Intriguingly, P2X7 receptor immunoreactive protein levels in the hippocampal CA1 region were profoundly suppressed by E2 treatment. We next examined whether E2 could regulate gene expression of the P2X7 receptor in the hippocampus at various time points after GCI. As shown in Figure 5(c), P2X7 receptor mRNA levels were significantly increased (1.5–2-fold) in the hippocampus from days 1, 3, and 7 after GCI, as compared to sham controls. E2 treatment had no effect upon the P2X7 receptor mRNA levels at day 1 after GCI but significantly attenuated its expression at days 3 and 7 after GCI. We also examined whether E2 could regulate another upstream regulator of NLRP3, thioredoxin interacting protein (TXNIP). Following increased production of reactive oxygen species (such as what occurs after ischemic injury), TXNIP is released from its complex with thioredoxin (TRX) and can bind to the LRR region of NLRP3 and activate it [52, 53]. As shown in Supplementary Figure 3A, immunofluorescence staining revealed that TXNIP expression was increased in rat hippocampal CA1 region at 7 days after GCI and that this expression is robustly suppressed by E2 treatment. Quantification of intensity of TXNIP for all images from all animals is shown in Supplementary Figure 3B. As shown in Supplementary Figure 3C, mRNA levels of TXNIP were also increased after GCI and significantly suppressed by E2 treatment at days 3 and 7 after GCI. These results suggest that E2 may potentially regulate the inflammasome complex activation via its ability to suppress expression of the NLRP3 inflammasome upstream regulators, P2X7 receptor and TXNIP.

### 3.4. The Estrogen Receptor Coregulator, PELP1, Is Essential for the Ability of Estradiol to Regulate the NLRP3 Inflammasome after GCI

**Figure 6:**
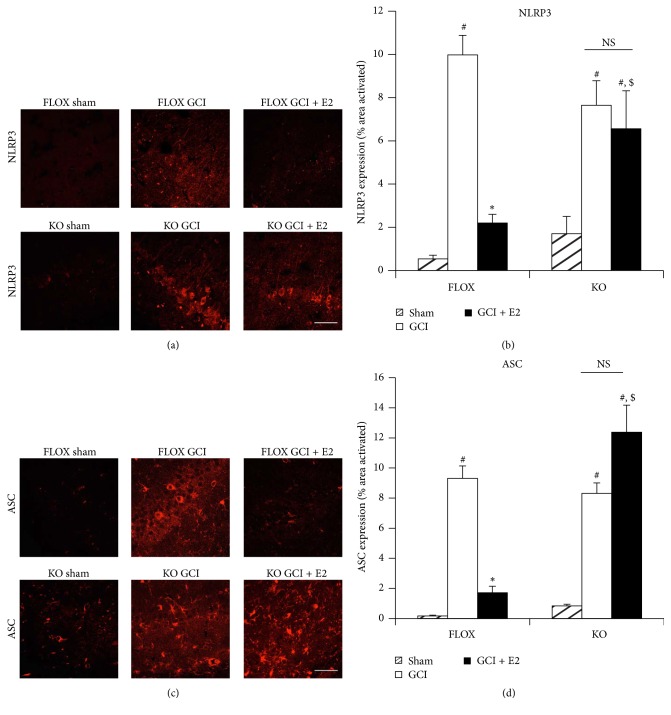
The ability of E2 to attenuate expression of NLRP3 and ASC after GCI is lost in PELP1 forebrain-specific knockout mice. (a and c) Representative confocal images show that NLRP3 and ASC are induced in hippocampal CA1 region at day 6 after GCI in FLOX control as well as PELP1 knockout mice. The expression of these markers is suppressed by E2 in the FLOX control mice but not in the PELP1 KO mice sections (KO = PELP1 knockout) (magnification = 40x, scale bar = 50 *μ*m). (b and d) Quantification of fluorescence intensity using MATLAB software shows a statistically significant suppression of all of these proteins by E2 in FLOX but not in KO mice (^#^ *p* < 0.05, sham versus GCI; ^*∗*^ *p* < 0.05, GCI versus GCI + E2; ^$^ *p* < 0.05, FLOX GCI + E2 versus KO GCI + E2; NS, not significant, KO GCI versus KO GCI + E2) (*n* = 5-6 animals/group).

To enhance understanding of the mechanisms involved in the E2 anti-inflammatory effects after GCI, we examined the role of the ER coregulator, PELP1. Prior work by our group using a PELP1 forebrain-specific KO (PELP1 FBKO) mouse model that we generated revealed that PELP1 is required for E2-induced rapid and genomic signaling, as well as the neuroprotective and cognitive-enhancing effects of E2 in the hippocampus after GCI [16]. Moreover, RNA-seq data comparing hippocampal gene expression in E2-treated FLOX control and PELP1 FBKO mice at 24 hours after GCI revealed that a number of inflammatory pathway genes were upregulated in the PELP1 FBKO mouse [16]. We therefore examined whether the ability of E2 to suppress expression of NLRP3 inflammasome pathway factors in the hippocampal CA1 region after GCI required PELP1 mediation. We utilized FLOX control and PELP1 FBKO mice and examined E2 regulation of NLRP3 inflammasome expression at 6 days after GCI, as this time point showed robust microglia activation and neuronal loss after GCI [16]. Representative photomicrographs of immunofluorescent staining results for NLRP3 and ASC are shown in Figures 6(a) and 6(c), respectively, while quantification of the results from all images from all animals for intensity using MATLAB is shown in Figures 6(b) and 6(d). As shown in Figures 6(a)–6(d), immunofluorescence staining of hippocampal CA1 region sections revealed that NLRP3 and ASC were markedly elevated at day 6 after GCI in FLOX control mice, and that E2 suppressed this effect. Intriguingly, the ability of E2 to suppress elevation of NLRP3 and ASC in the hippocampus was lost in PELP1 FBKO mice (Figures 6(a)–6(d)).

**Figure 7:**
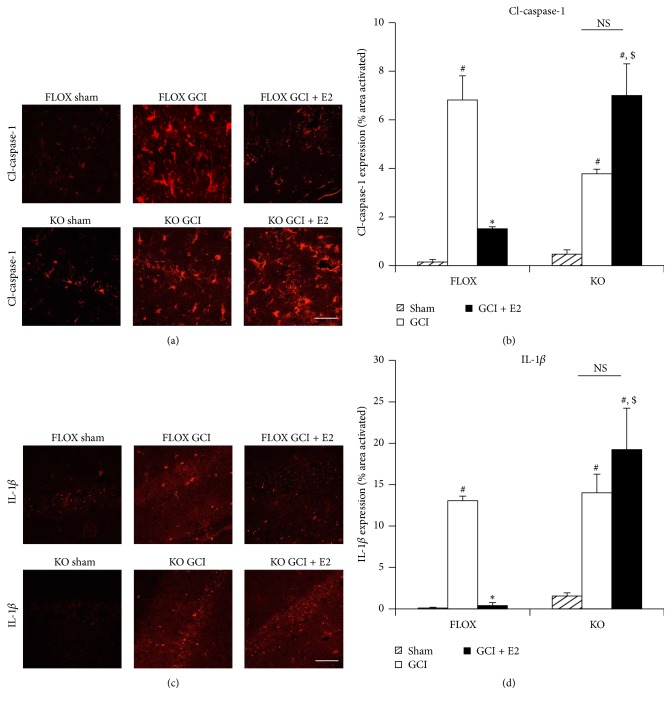
The ability of E2 to attenuate expression of cleaved caspase-1 and IL-1*β* after GCI is lost in PELP1 forebrain-specific knockout mice. (a and c) Representative confocal images show that cleaved caspase-1 and IL-1*β* are induced in hippocampal CA1 region at day 6 after GCI in FLOX as well as PELP1 knockout mice. The expression of these markers is suppressed by E2 in the FLOX control mice but not in the PELP1 KO mice sections (KO = PELP1 knockout) (magnification = 40x, scale bar = 50 *μ*m). (b and d) Quantification of fluorescence intensity using MATLAB software shows a statistically significant suppression of all of these proteins by E2 in FLOX but not in KO mice (^#^ *p* < 0.05, sham versus GCI; ^*∗*^ *p* < 0.05, GCI versus GCI + E2; ^$^ *p* < 0.05, FLOX GCI + E2 versus KO GCI + E2; NS, not significant, KO GCI versus KO GCI + E2) (*n* = 5-6 animals/group).

We next examined the downstream products of the NLRP3 inflammasome pathway, “active” cleaved caspase-1 and IL-1*β*, using immunofluorescence staining of hippocampal sections. Representative photomicrographs of immunofluorescent staining results for “active” cleaved caspase-1 and IL-1*β* are shown in Figures 7(a) and 7(c), respectively, while quantification of the results from all images from all animals for intensity using MATLAB is shown in Figures 7(b) and 7(d). As shown in Figures 7(a)–7(d), immunoreactive protein levels of “active” cleaved caspase-1 and IL-1*β* were significantly enhanced in the hippocampal CA1 region at 6 days after GCI in FLOX control mice, and E2 strongly suppressed the elevation of both of these factors. Further, the ability of E2 to suppress elevation of “active” cleaved caspase-1 and IL-1*β* in the hippocampus was lost in PELP1 FBKO mice (Figures 7(a)–7(d)).

**Figure 8:**
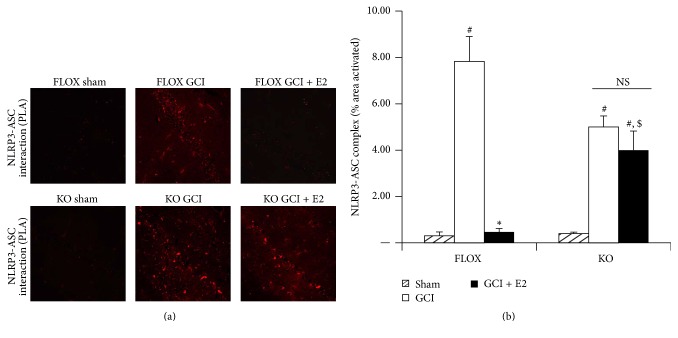
The ability of E2 to suppress NLRP3-ASC complex formation after GCI is lost in PELP1 forebrain-specific knockout mice. (a) Representative confocal images of Duolink* in situ* co-IP show that ASC-NLRP3 complex is formed in hippocampal CA1 region at day 6 after GCI. This complex formation is robustly suppressed by E2 in the FLOX but not in KO (KO = PELP1 knockout) (magnification = 40x, scale bar = 50 *μ*m). (b) MATLAB analysis of the images shows a statistically significant suppression of complex formation by E2 in FLOX but not in KO mice (^#^ *p* < 0.05, sham versus GCI; ^*∗*^ *p* < 0.05, GCI versus GCI + E2; ^$^ *p* < 0.05, FLOX GCI + E2 versus KO GCI + E2; NS, not significant, KO GCI versus KO GCI + E2) (*n* = 5-6 animals/group).

We next used the* in situ* proximity ligation assay to determine whether the ability of E2 to suppress NLRP3-ASC complex formation after GCI is compromised in PELP1 FBKO mice. Representative photomicrographs of the proximity ligation assay results are shown in Figure 8(a), while quantification of the results from all images from all animals for intensity of using MATLAB is shown in Figure 8(b). As shown in Figure 8, FLOX control mice demonstrated a significantly increased NLRP3-ASC complex formation in the hippocampal CA1 region at 6 days after GCI as detected by the proximity ligation assay, and E2 treatment completely attenuated the complex formation. However, the ability of E2 to inhibit the GCI-induced NLRP3-ASC complex formation was lost in PELP1 FBKO mice (Figures 8(a) and 8(b)).

**Figure 9:**
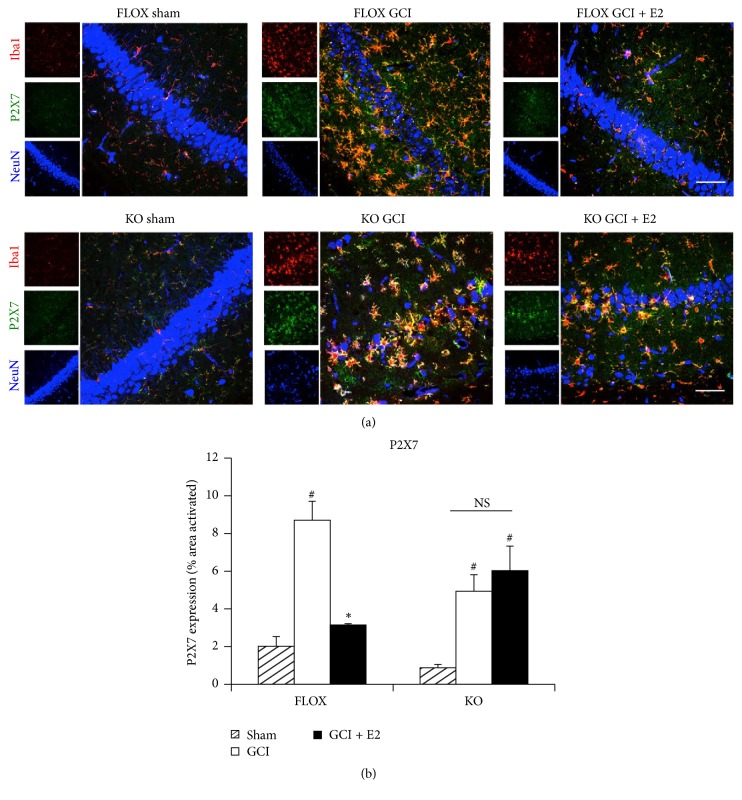
The ability of E2 to attenuate expression of P2X7 receptor after GCI is lost in PELP1 forebrain-specific knockout mice. (a) Representative confocal images stained for Iba1 (red), P2X7 receptor (green), and NeuN (blue) show the activation of microglia and increased expression of P2X7 receptor in the hippocampal CA1 region of young adult female FLOX control as well as PELP1 KO mice 6 days after GCI. The P2X7 receptor colocalized very strongly with microglia. This expression and microglial activation are suppressed by E2 treatment in FLOX control mice but not in PELP1 KO mice (KO = PELP1 knockout) (magnification = 40x, scale bar = 50 *μ*m). (b) MATLAB analysis of the images shows a statistically significant suppression of all of these proteins by E2 in FLOX control but not in KO mice (^#^ *p* < 0.05, sham versus GCI; ^*∗*^ *p* < 0.05, GCI versus GCI + E2; NS, not significant, KO GCI versus KO GCI + E2) (*n* = 5-6 animals/group).

Finally, we examined the effect of PELP1 deletion upon the ability of E2 to suppress expression of the P2X7 receptor in hippocampal CA1 region after GCI. Representative photomicrographs of triple immunofluorescent staining results for the P2X7 receptor, Iba1 (microglia marker), and NeuN (neuronal marker) are shown in Figure 9(a), while quantification of the results from all images from all animals for intensity using MATLAB is shown in Figure 9(b). As shown in Figures 9(a) and 9(b), triple immunofluorescence staining for the P2X7 receptor, Iba1 (microglia marker), and NeuN (neuronal marker) revealed that there was increased expression of the P2X7 receptor in the hippocampal CA1 region of FLOX control as well as PELP1 knockout mice after GCI. The P2X7 receptor immunoreactive signal colocalized with Iba1, indicating that the enhanced P2X7 expression occurred primarily in microglia. As was the case in the ovariectomized rat, P2X7 receptor expression was suppressed by E2 treatment in FLOX control mice that were subjected to GCI. The suppressive effect of E2 on P2X7 receptor expression required PELP1 mediation as evidenced by the loss of the suppressive effect of E2 in PELP1 FBKO mice (Figures 9(a) and 9(b)).

## 4. Discussion 

The current study provides several important findings. First, it demonstrates that the NLRP3 inflammasome is robustly activated in microglia and astrocytes in the hippocampal CA1 region of both the rat and mouse after GCI. Secondly, it demonstrates that low dose E2 treatment profoundly suppresses NLRP3 inflammasome activation in the hippocampus after GCI. Thirdly, it provides novel insight into the mechanisms of E2 anti-inflammatory effects by demonstrating that E2 suppresses induction of the upstream activators of the NLRP3 inflammasome (P2X7 and TXNIP) after GCI, and that the ER coregulator protein, PELP1, is essential for the anti-inflammatory actions of E2 in the hippocampus.

A unique strength of our study is that we used a novel genetic PELP1 forebrain-specific knockout model to elucidate the role of PELP1 in E2 anti-inflammatory actions. Based on the results of our study, we propose that E2 binds to ER and recruits the coregulator PELP1 to form an active transcriptional complex, which leads to attenuation of expression of the upstream inducers of the NLRP3 inflammasome, P2X7 and TXNIP in the hippocampus after GCI. Suppression of TXNIP and NLRP3 activation could also be due to E2 suppression of GCI-induced ROS generation, as we previously demonstrated that E2 profoundly suppresses NOX2 NADPH oxidase activity and superoxide induction after GCI [7]. Furthermore, in support of this possibility, NOX2 knockout mice have been shown to have a significantly reduced NLRP3 activation after FCI that correlated with reduced infarct size, reduced edema, and improved neurological outcome [38]. While we believe that E2 effects involve mediation by nuclear ER, we cannot rule out the possibility that extranuclear ER signaling could be involved as well, as we previously demonstrated that PELP1 also mediates extranuclear ER-mediated rapid signaling effects in the hippocampus after GCI [16].

While our study did not address which ER mediates E2 effects upon the NLRP3 inflammasome after GCI, PELP1 can interact with and mediate the effects of both ER-*α* and ER-*β* in various cells throughout the body [54]. Intriguingly, both ER-*α* and ER-*β* have been implicated to mediate anti-inflammatory actions of E2 in the brain. Most relevant to our study, ER-*β* antisense oligonucleotide knockdown has been reported to attenuate E2 suppression of ASC, caspase-1, and IL-1*β* after GCI [47]. This study did not examine which inflammasome mediated the increase in IL-1*β* and was regulated by E2, but our work provides strong evidence that the NLRP3 inflammasome is activated in the hippocampus after GCI and strongly regulated by E2. ER-*α* has also been implicated in mediating some of the anti-inflammatory effects of E2 in the brain. For instance, Vegeto et al. [28] found that the ability of E2 to suppress lipopolysaccharide-induced microglial activation and monocyte recruitment in the brain is lost in ER-*α* knockout mice. However, this study did not assess the role for ER-*α* in inflammasome regulation. Finally, to our knowledge, no study has examined the role of GPER1 in the regulation of inflammasome activation in the brain. Studies are ongoing in our laboratory to further address this issue.

Of significant note, our study found that NLRP3 pathway factors were induced in both microglia and astrocytes after GCI. A similar multicell induction of NLRP3 pathway factors has been reported previously in the cerebral cortex after focal cerebral ischemia [55] or traumatic brain injury [22], demonstrating that NLPR3 factors are expressed in neurons, astrocytes, and microglia after brain injury. Likewise, expression of P2X7 receptors has been reported in multiple brain cell types, including microglia, astrocytes, and neurons [56–59], and is increased after cerebral ischemia [60–62]. Thus, it appears that multiple cell types can express inflammasomes and be involved in neuroinflammation. While less is known about the role of NLRP3 inflammasome activation in astrocytes as compared to microglia, recent studies have found that astrocytes can express NLRP3 and ASC and display caspase-1 cleavage and release of IL-1*β* after induction with inflammatory agents, A*β*, or* in vitro* oxygen/glucose deprivation [36, 37, 63]. This suggests that inflammasome activation in astrocytes may also contribute to neuroinflammation after brain injury. In contrast, NLRP1 and Aim2 expression has been primarily reported in neurons in the ischemic or injured brain [64–66].

Finally, previous work by our laboratory and others has shown that E2 is profoundly neuroprotective and improves functional outcome after GCI and FCI [5, 9–11, 16, 67]. Based on our current results, we propose that E2 inhibition of NLRP3 inflammasome activation is an important contributing mechanism for E2 beneficial effects on neuronal survival and functional outcome after GCI. In support of this possibility, NLRP3 knockout mice have been shown to have significantly reduced infarct size after FCI, suggesting that NLRP3 inflammasome activation contributes to neuronal damage and cell death after cerebral ischemia [38]. Furthermore, intravenous immunoglobulin has been shown to suppress NLRP3 inflammasome-mediated neuronal cell death after ischemic stroke and to improve neurological outcome [40]. Finally, caspase-1 knockout mice, as well as caspase-1 inhibitor- or dominant negative-treated mice, have been all reported to have significantly decreased neuronal cell death and brain deficits following cerebral ischemia [68–71]. Collectively, these findings suggest that NLRP3 inflammasome activation and resultant neuroinflammation contribute significantly to the neuronal cell death and functional deficits that occur after cerebral ischemia, and that E2 inhibition of NLRP3 inflammasome activation may contribute significantly to its beneficial protective effects following cerebral ischemia.

In conclusion, the results of our study demonstrate that NLRP3 inflammasome activation is strongly increased in the hippocampus after GCI, and that E2 strongly suppresses both expression and activation of the NLRP3 inflammasome. The effects of E2 require the ER-coregulator, PELP1, and involve attenuation of P2X7 and TXNIP, two well-known upstream inducers of NLRP3 inflammasome activation. These findings provide new insight into the anti-inflammatory effects of E2 in the brain and suggest that therapeutic targeting of the NLRP3 inflammasome for inhibition by E2 analogues or NLRP3 inflammasome selective inhibitors may have efficacy in the treatment of GCI, as well as other neurodegenerative disorders that involve NLRP3 inflammasome activation and neuroinflammation.

## Supplementary Material

Antigen or recombinant protein preabsorption essentially completely abolished staining for the NLRP3 inflammasome molecules, demonstrating specificity of the antibodies.

## Figures and Tables

**Table 1:** 

Gene	Forward primer	Reverse primer
NLRP3	5′ AGAAGCTGGGGTTGGTGAATT 3′	5′ GTTGTCTAACTCCAGCATCTG 3′
ASC	5′ CCCATAGACCTCACTGATAAAC 3′	5′ AGAGCATCCAGCAAACCA 3′
Caspase-1	5′ AGGAGGGAATATGTGGG 3′	5′ AACCTTGGGCTTGTCTT 3′
IL-1*β*	5′ TGACCCATGTGAGCTGAAAG 3′	5′ AGGGATTTTGTCGTTGCTTG 3′
TXNIP	5′ CTGATGGAGGCACAGTGAGA 3′	5′ CTCGGGTGGAGTGCTTAG 3′
P2rX7	5′ CTACTCTTCGGTGGGGGCTT 3′	5′ AACCCTGGTCAGAATGGCAC 3′
CypA	5′ TATCTGCACTGCCAAGACTGAGTG 3′	5′ CTTCTTGCTGGTCTTGCCATTCC 3′

## Abbreviations

ASC: Apoptosis associated speck like protein
E2: 17*β*-Estradiol
ER-*α*: Estrogen receptor alpha
ER-*β*: Estrogen receptor-beta
FCI: Focal cerebral ischemia
GCI: Global cerebral ischemia
IL-1*β*: Interleukin-1beta
NLRP3: NOD-like receptor with a pyrin domain-3
PELP1: Proline-, glutamic acid-, and leucine-rich protein 1
TXNIP: Thioredoxin interacting protein.

## References

[1] Lebesgue D., Chevaleyre V., Zukin R. S., Etgen A. M. (2009). Estradiol rescues neurons from global ischemia-induced cell death: multiple cellular pathways of neuroprotection. *Steroids*.

[2] Simpkins J. W., Perez E., Xiaofei Wang, Shaohua Yang, Yi Wen, Singh M. (2009). The potential for estrogens in preventing Alzheimer's disease and vascular dementia. *Therapeutic Advances in Neurological Disorders*.

[3] Simpkins J. W., Green P. S., Gridley K. E., Singh M., De Fiebre N. C., Rajakumar G. (1997). Role of estrogen replacement therapy in memory enhancement and the prevention of neuronal loss associated with Alzheimer's disease. *The American Journal of Medicine*.

[4] Engler-Chiurazzi E. B., Brown C., Povroznik J., Simpkins J. (2016). Estrogens as neuroprotectants: estrogenic actions in the context of cognitive aging and brain injury. *Progress in Neurobiology*.

[5] Simpkins J. W., Rajakumar G., Zhang Y.-Q. (1997). Estrogens may reduce mortality and ischemic damage caused by middle cerebral artery occlusion in the female rat. *Journal of Neurosurgery*.

[6] Tang H., Zhang Q., Yang L. (2014). GPR30 mediates estrogen rapid signaling and neuroprotection. *Molecular and Cellular Endocrinology*.

[7] Zhang Q.-G., Raz L., Wang R. (2009). Estrogen attenuates ischemic oxidative damage via an estrogen receptor *α*-mediated inhibition of NADPH oxidase activation. *The Journal of Neuroscience*.

[8] Yang L.-C., Zhang Q.-G., Zhou C.-F. (2010). Extranuclear estrogen receptors mediate the neuroprotective effects of estrogen in the rat hippocampus. *PLoS ONE*.

[9] Brann D. W., Dhandapani K., Wakade C., Mahesh V. B., Khan M. M. (2007). Neurotrophic and neuroprotective actions of estrogen: basic mechanisms and clinical implications. *Steroids*.

[10] Dubal D. B., Kashon M. L., Pettigrew L. C. (1998). Estradiol protects against ischemic injury. *Journal of Cerebral Blood Flow and Metabolism*.

[11] Jover T., Tanaka H., Calderone A. (2002). Estrogen protects against global ischemia-induced neuronal death and prevents activation of apoptotic signaling cascades in the hippocampal CA1. *Journal of Neuroscience*.

[12] Bourque M., Morissette M., Di Paolo T. (2015). Neuroprotection in Parkinsonian-treated mice via estrogen receptor *α* activation requires G protein-coupled estrogen receptor 1. *Neuropharmacology*.

[13] Prossnitz E. R., Barton M. (2014). Estrogen biology: new insights into GPER function and clinical opportunities. *Molecular and Cellular Endocrinology*.

[14] Arevalo M.-A., Azcoitia I., Garcia-Segura L. M. (2015). The neuroprotective actions of oestradiol and oestrogen receptors. *Nature Reviews Neuroscience*.

[15] Vadlamudi R. K., Wang R.-A., Mazumdar A. (2001). Molecular cloning and characterization of PELP1, a novel human co-regulator of estrogen receptor *α*. *The Journal of Biological Chemistry*.

[16] Sareddy G. R., Zhang Q., Wang R. (2015). Proline-, glutamic acid-, and leucine-rich protein 1 mediates estrogen rapid signaling and neuroprotection in the brain. *Proceedings of the National Academy of Sciences of the United States of America*.

[17] Sareddy G. R., Vadlamudi R. K. (2016). PELP1: structure, biological function and clinical significance. *Gene*.

[18] Khan M. M., Hadman M., Wakade C. (2005). Cloning, expression, and localization of MNAR/PELP1 in rodent brain: colocalization in estrogen receptor-*α*- but not in gonadotropin-releasing hormone-positive neurons. *Endocrinology*.

[19] Khan M. M., Hadman M., De Sevilla L. M. (2007). Cloning, distribution, and colocalization of MNAR/PELP1 with glucocorticoid receptors in primate and nonprimate brain. *Neuroendocrinology*.

[20] Vadlamudi R. K., Wang R.-A., Mazumdar A. (2001). Molecular cloning and characterization of PELP1, a novel human coregulator of estrogen receptor *α*. *Journal of Biological Chemistry*.

[21] Heneka M. T., Kummer M. P., Stutz A. (2013). NLRP3 is activated in Alzheimer's disease and contributes to pathology in APP/PS1 mice. *Nature*.

[22] Liu H.-D., Li W., Chen Z.-R. (2013). Expression of the NLRP3 inflammasome in cerebral cortex after traumatic brain injury in a rat model. *Neurochemical Research*.

[23] Singhal G., Jaehne E. J., Corrigan F., Toben C., Baune B. T. (2014). Inflammasomes in neuroinflammation and changes in brain function: a focused review. *Frontiers in Neuroscience*.

[24] Vegeto E., Benedusi V., Maggi A. (2008). Estrogen anti-inflammatory activity in brain: a therapeutic opportunity for menopause and neurodegenerative diseases. *Frontiers in Neuroendocrinology*.

[25] Dang J., Mitkari B., Kipp M., Beyer C. (2011). Gonadal steroids prevent cell damage and stimulate behavioral recovery after transient middle cerebral artery occlusion in male and female rats. *Brain, Behavior, and Immunity*.

[26] Brown C. M., Mulcahey T. A., Filipek N. C., Wise P. M. (2010). Production of proinflammatory cytokines and chemokines during neuroinflammation: novel roles for estrogen receptors *α* and *β*. *Endocrinology*.

[27] Vegeto E., Belcredito S., Ghisletti S., Meda C., Etteri S., Maggi A. (2006). The endogenous estrogen status regulates microglia reactivity in animal models of neuroinflammation. *Endocrinology*.

[28] Vegeto E., Belcredito S., Etteri S. (2003). Estrogen receptor-alpha mediates the brain antiinflammatory activity of estradiol. *Proceedings of the National Academy of Sciences of the United States of America*.

[29] Martinon F., Burns K., Tschopp J. (2002). The inflammasome: a molecular platform triggering activation of inflammatory caspases and processing of proIL-*β*. *Molecular Cell*.

[30] Guo H., Callaway J. B., Ting J. P.-Y. (2015). Inflammasomes: mechanism of action, role in disease, and therapeutics. *Nature Medicine*.

[31] Walsh J. G., Muruve D. A., Power C. (2014). Inflammasomes in the CNS. *Nature Reviews Neuroscience*.

[32] De Rivero Vaccari J. P., Dietrich W. D., Keane R. W. (2014). Activation and regulation of cellular inflammasomes: gaps in our knowledge for central nervous system injury. *Journal of Cerebral Blood Flow and Metabolism*.

[33] Leo Bours M. J., Dagnelie P. C., Giuliani A. L., Wesselius A., Di Virgilio F. (2011). P2 receptors and extracellular ATP: a novel homeostatic pathway in inflammation. *Frontiers in Bioscience (Scholar Edition)*.

[34] Martinon F., Mayor A., Tschopp J. (2009). The inflammasomes: guardians of the body. *Annual Review of Immunology*.

[35] Freeman L. C., Ting J. P.-Y. (2016). The pathogenic role of the inflammasome in neurodegenerative diseases. *Journal of Neurochemistry*.

[36] Jian Z., Ding S., Deng H. (2016). Probenecid protects against oxygen-glucose deprivation injury in primary astrocytes by regulating inflammasome activity. *Brain Research*.

[37] Alfonso-Loeches S., Ureña-Peralta J. R., Morillo-Bargues M. J., La Cruz J. O.-D., Guerri C. (2014). Role of mitochondria ROS generation in ethanol-induced NLRP3 inflammasome activation and cell death in astroglial cells. *Frontiers in Cellular Neuroscience*.

[38] Yang F., Wang Z., Wei X. (2014). NLRP3 deficiency ameliorates neurovascular damage in experimental ischemic stroke. *Journal of Cerebral Blood Flow and Metabolism*.

[39] Lammerding L., Slowik A., Johann S., Beyer C., Zendedel A. (2016). Poststroke inflammasome expression and regulation in the peri-infarct area by gonadal steroids after transient focal ischemia in the rat brain. *Neuroendocrinology*.

[40] Yang-Wei Fann D., Lee S.-Y., Manzanero S. (2013). Intravenous immunoglobulin suppresses NLRP1 and NLRP3 inflammasome-mediated neuronal death in ischemic stroke. *Cell Death and Disease*.

[41] Fann D. Y.-W., Santro T., Manzanero S. (2014). Intermittent fasting attenuates inflammasome activity in ischemic stroke. *Experimental Neurology*.

[42] Tong Y., Ding Z. H., Zhan F. X. (2015). The NLRP3 inflammasome and stroke. *International Journal of Clinical and Experimental Medicine*.

[43] Wang X., Li R., Wang X., Fu Q., Ma S. (2015). Umbelliferone ameliorates cerebral ischemia-reperfusion injury via upregulating the PPAR gamma expression and suppressing TXNIP/NLRP3 inflammasome. *Neuroscience Letters*.

[44] Li Y., Li J., Li S. (2015). Curcumin attenuates glutamate neurotoxicity in the hippocampus by suppression of ER stress-associated TXNIP/NLRP3 inflammasome activation in a manner dependent on AMPK. *Toxicology and Applied Pharmacology*.

[45] Denes A., Coutts G., Lénárt N. (2015). AIM2 and NLRC4 inflammasomes contribute with ASC to acute brain injury independently of NLRP3. *Proceedings of the National Academy of Sciences*.

[46] Slowik A., Beyer C. (2015). Inflammasomes are neuroprotective targets for sex steroids. *Journal of Steroid Biochemistry and Molecular Biology*.

[47] De Rivero Vaccari J. P., Patel H. H., Brand F. J., Perez-Pinzon M. A., Bramlett H. M., Raval A. P. (2016). Estrogen receptor beta signaling alters cellular inflammasomes activity after global cerebral ischemia in reproductively senescence female rats. *Journal of Neurochemistry*.

[48] Pulsinelli W. A., Brierley J. B. (1979). A new model of bilateral hemispheric ischemia in the unanesthetized rat. *Stroke*.

[49] Langnaese K., John R., Schweizer H., Ebmeyer U., Keilhoff G. (2008). Selection of reference genes for quantitative real-time PCR in a rat asphyxial cardiac arrest model. *BMC Molecular Biology*.

[50] Kozlowski C., Weimer R. M. (2012). An automated method to quantify microglia morphology and application to monitor activation state longitudinally in vivo. *PLoS ONE*.

[51] Gombault A., Baron L., Couillin I. (2012). ATP release and purinergic signaling in NLRP3 inflammasome activation. *Frontiers in Immunology*.

[52] Zhou R., Tardivel A., Thorens B., Choi I., Tschopp J. (2010). Thioredoxin-interacting protein links oxidative stress to inflammasome activation. *Nature Immunology*.

[53] Minutoli L., Puzzolo D., Rinaldi M. (2016). ROS-mediated NLRP3 inflammasome activation in brain, heart, kidney, and testis ischemia/reperfusion injury. *Oxidative Medicine and Cellular Longevity*.

[54] Brann D. W., Zhang Q.-G., Wang R.-M., Mahesh V. B., Vadlamudi R. K. (2008). PELP1-A novel estrogen receptor-interacting protein. *Molecular and Cellular Endocrinology*.

[55] Lammerding L., Slowik A., Johann S., Beyer C., Zendedel A. (2016). Post-stroke inflammasome expression and regulation in the peri-infarct area by gonadal steroids after transient focal ischemia in the rat brain. *Neuroendocrinology*.

[56] Yu Y., Ugawa S., Ueda T. (2008). Cellular localization of P2X7 receptor mRNA in the rat brain. *Brain Research*.

[57] Collo G., Neidhart S., Kawashima E., Kosco-Vilbois M., North R. A., Buell G. (1997). Tissue distribution of the P2X7 receptor. *Neuropharmacology*.

[58] Nagasawa K., Escartin C., Swanson R. A. (2009). Astrocyte cultures exhibit P2X7 receptor channel opening in the absence of exogenous ligands. *Glia*.

[59] Ohishi A., Keno Y., Marumiya A. (2016). Expression level of P2X7 receptor is a determinant of ATP-induced death of mouse cultured neurons. *Neuroscience*.

[60] Li X.-J., He R.-F., Li S., Li X.-J., Li D.-L. (2012). Effects of progesterone on learning and memory and P2X7 receptor expression in the hippocampus after global cerebral ischemia/ reperfusion injury in rats. *Zhongguo Ying Yong Sheng Li Xue Za Zhi*.

[61] Lu Y.-M., Tao R.-R., Huang J.-Y. (2012). P2X 7 signaling promotes microsphere embolism-triggered microglia activation by maintaining elevation of Fas ligand. *Journal of Neuroinflammation*.

[62] Melani A., Amadio S., Gianfriddo M. (2006). P2X7 receptor modulation on microglial cells and reduction of brain infarct caused by middle cerebral artery occlusion in rat. *Journal of Cerebral Blood Flow and Metabolism*.

[63] Couturier J., Stancu I.-C., Schakman O. (2016). Activation of phagocytic activity in astrocytes by reduced expression of the inflammasome component ASC and its implication in a mouse model of Alzheimer disease. *Journal of Neuroinflammation*.

[64] Wang Y.-C., Li W.-Z., Wu Y. (2015). Acid-sensing ion channel 1a contributes to the effect of extracellular acidosis on NLRP1 inflammasome activation in cortical neurons. *Journal of Neuroinflammation*.

[65] Adamczak S. E., De Rivero Vaccari J. P., Dale G. (2014). Pyroptotic neuronal cell death mediated by the AIM2 inflammasome. *Journal of Cerebral Blood Flow and Metabolism*.

[66] Wang X., Chu G., Yang Z. (2015). Ethanol directly induced HMGB1 release through NOX2/NLRP1 inflammasome in neuronal cells. *Toxicology*.

[67] Zhang Q.-G., Wang R., Khan M., Mahesh V., Brann D. W. (2008). Role of Dickkopf-1, an antagonist of the Wnt/*β*-catenin signaling pathway, in estrogen-induced neuroprotection and attenuation of tau phosphorylation. *Journal of Neuroscience*.

[68] Friedlander R. M., Gagliardini V., Hara H. (1997). Expression of a dominant negative mutant of interleukin-1*β* converting enzyme in transgenic mice prevents neuronal cell death induced by trophic factor withdrawal and ischemic brain injury. *Journal of Experimental Medicine*.

[69] Hara H., Friedlander R. M., Gagliardini V. (1997). Inhibition of interleukin 1*β* converting enzyme family proteases reduces ischemic and excitotoxic neuronal damage. *Proceedings of the National Academy of Sciences of the United States of America*.

[70] Rabuffetti M., Sciorati C., Tarozzo G., Clementi E., Manfredi A. A., Beltramo M. (2000). Inhibition of caspase-1-like activity by Ac-Tyr-Val-Ala-Asp-chloromethyl ketone induces long-lasting neuroprotection in cerebral ischemia through apoptosis reduction and decrease of proinflammatory cytokines. *Journal of Neuroscience*.

[71] Schielke G. P., Yang G.-Y., Shivers B. D., Betz A. L. (1998). Reduced ischemic brain injury in interleukin-1*β* converting enzyme- deficient mice. *Journal of Cerebral Blood Flow and Metabolism*.

