# Supplementary figures and images for: NLRP3 Inflammasome Activation in the Brain after Global Cerebral Ischemia and Regulation by 17β-Estradiol
Source: Oxid Med Cell Longev. 2016 Oct 23;2016:8309031. doi: 10.1155/2016/8309031 (PMC5097821; doi:10.1155/2016/8309031)

# Supplementary Figure 1

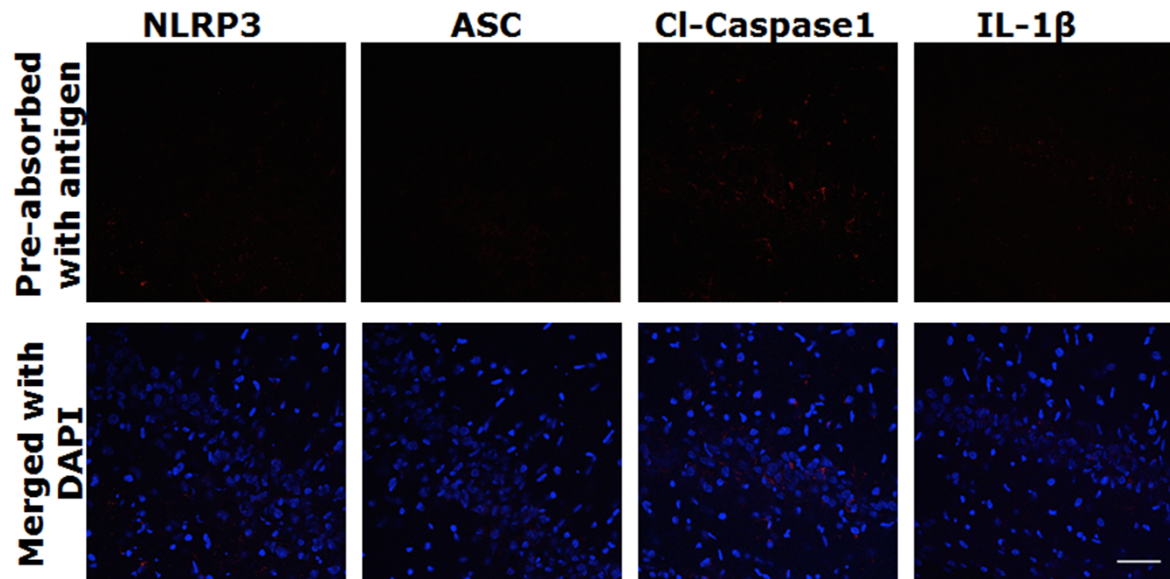

## Supplementary Figure 2

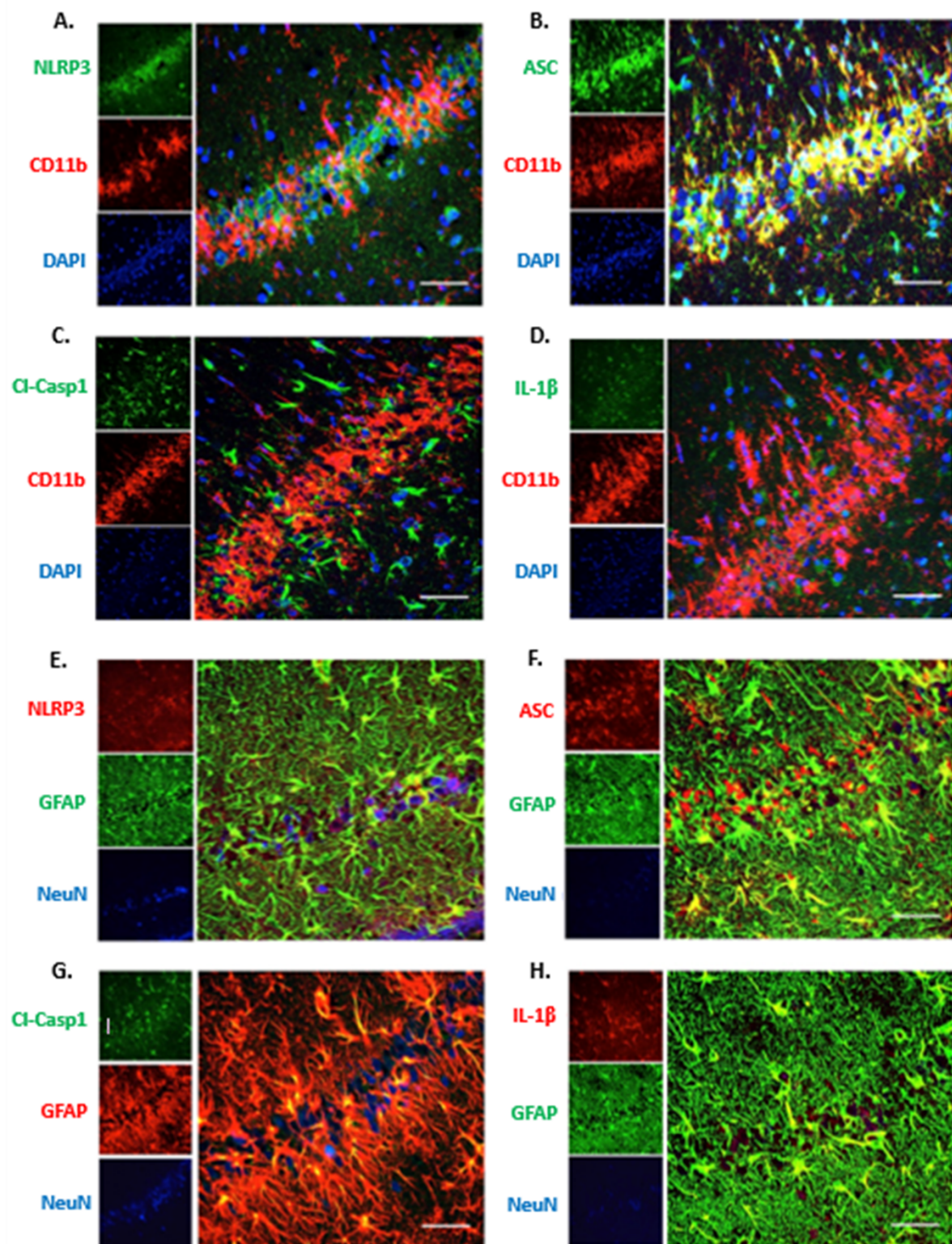

## Supplementary Figure 3

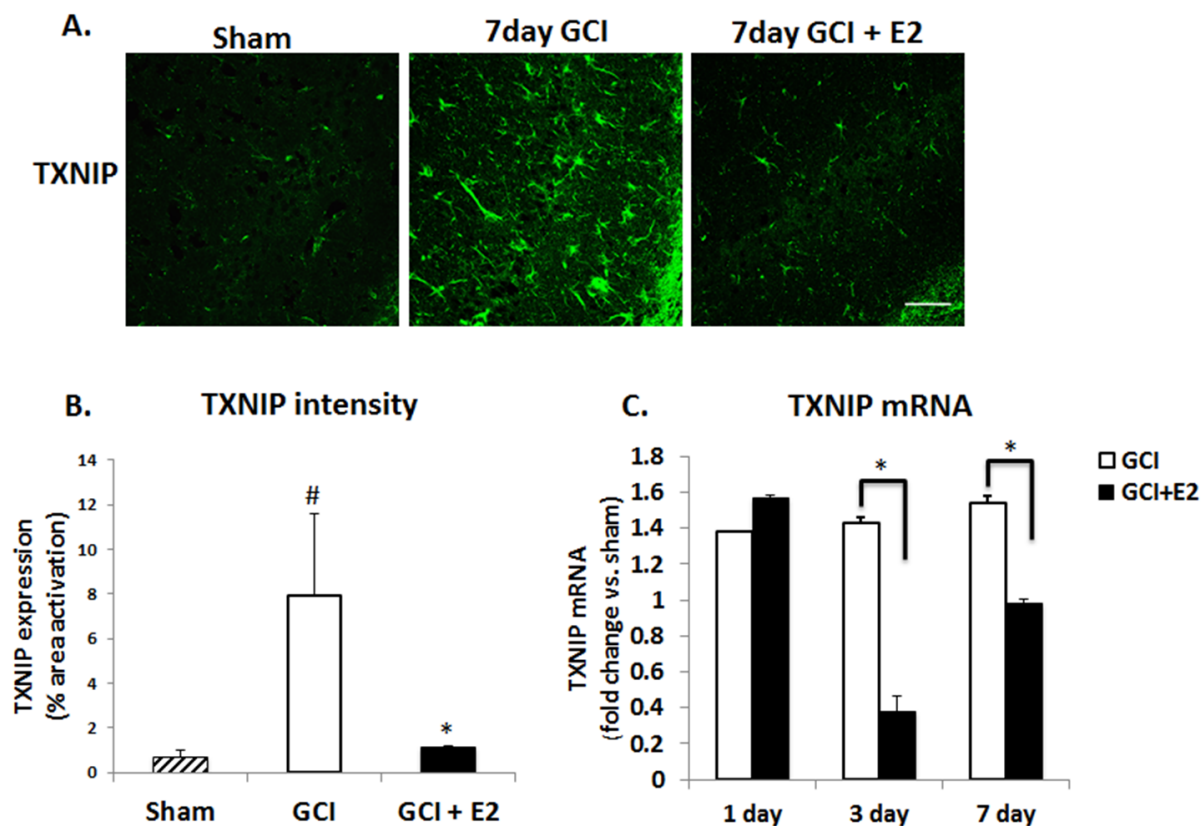

Supplement: Supplementary file 1 — Antigen or recombinant protein preabsorption essentially completely abolished staining for the NLRP3 inflammasome molecules, demonstrating specificity of the antibodies. [file 8309031.f1.pdf]
